# Pemetrexed-Platinum With or Without Bevacizumab for Chinese Chemo-Naive Advanced Lung Adenocarcinoma Patients: A Real-World Study

**DOI:** 10.3389/fphar.2021.649222

**Published:** 2021-05-07

**Authors:** Xin Li, Jie Huang, Yao Qiu, Qianyun Zhang, Shaoyu Yang, Kan Wu, Jiaoli Wang, Limin Wang, Jian Ye, Shenglin Ma, Bing Xia, Xueqin Chen

**Affiliations:** ^1^ Department of Thoracic Oncology, Key Laboratory of Clinical Cancer Pharmacology and Toxicology Research of Zhejiang Province, Affiliated Hangzhou First People’s Hospital, Zhejiang University School of Medicine, Hangzhou, China; ^2^ Department of Thoracic Oncology, Hangzhou Cancer Hospital, Zhejiang University School of Medicine, Hangzhou, China; ^3^ Department of Thoracic Oncology, Nanjing Medical University Affiliated Hangzhou Hospital, Hangzhou, China; ^4^ Department of Respiratory Disease, Affiliated Hangzhou First People’s Hospital, Zhejiang University School of Medicine, Hangzhou, China; ^5^ Cancer Center, Zhejiang University, Hangzhou, China

**Keywords:** bevacizumab, pemetrexed, platinum, lung adenocarcinoma, adrenal metastasis

## Abstract

Despite recent advances in the treatment of advanced non–small-cell lung cancer (NSCLC), bevacizumab plus platinum–based doublet chemotherapy remains a commonly used first-line regimen. This study was conducted to compare the efficacy and safety of pemetrexed–platinum with or without bevacizumab in Chinese chemo-naive advanced lung adenocarcinoma patients in a real-world setting. We retrospectively collected 100 patients who received pemetrexed–platinum with or without bevacizumab (PP, *n* = 46; Bev+PP, *n* = 54) until disease progression or unacceptable toxicity. Clinical characteristics of patients were balanced, except for the proportion of stage IV b+c (Bev+PP and PP: 67.4 vs. 37.0%, *p* = 0.0066). Bev+PP significantly improved the objective response rate (ORR, 65 vs. 30%, *p* = 0.0004) and progression-free survival (PFS, 7.4 vs. 6.8 months, *p* = 0.009), but not overall survival (OS, 17.5 vs. 15.0 months, *p* = 0.553) compared with PP. Treatment (*p* = 0.001), gender (*p* = 0.008), adrenal metastasis (*p* = 0.001), and liver metastasis (*p* = 0.013) were independent risk factors for PFS. Patients with adrenal metastasis tended to be at the highest risk of not benefiting from bevacizumab addition (HR [95% CI]: 2.244 [0.6495–7.753]). The safety profile was acceptable, and grade ≥3 toxicity occurred similarly. This study showed that pemetrexed–platinum plus bevacizumab was effective compared to chemotherapy alone in Chinese patients with advanced NSCLC.

## Introduction

Lung cancer is the most common cancer in the world (Ferlay et al., 2019), and China is also faced with a heavy burden of lung cancer (Cao et al., 2020; Yang et al., 2020) which is linked to tobacco smoking, outdoor air pollution, household air pollution, etc. (Vermeulen et al., 2019; Guan et al., 2020). The rapid development of the pharmaceutical industry and molecular biology research, especially on the tumor-associated immune microenvironment, has resulted in a variety of treatment options for lung adenocarcinoma patients including small-molecule tyrosine kinase inhibitors (TKIs) that target the EGFR/ALK/ROS1 (EAR) gene (Pakkala and Ramalingam, 2018) and immune checkpoint inhibitors that block the PD1/PD-L1 and B7/CTLA4 pathways (Bansal et al., 2016). However, for patients lacking the EAR mutations and PD-L1 expression, or in patients where these therapeutic strategies fail, platinum-based chemotherapy regimens have been commonly used in clinical practice.

Bevacizumab is a widely researched monoclonal antibody that inhibits VEGF-A and has been approved in combination with chemotherapy for the treatment of chemo-naive non–small-cell lung cancer patients (Assoun et al., 2017; Garcia et al., 2020). Previous studies have shown that VEGF-A functions as a high-risk factor dampening the prognosis of lung cancer patients (Hu et al., 2013). Serum VEGF attenuates the efficacy of platinum-based chemotherapy in non–small-cell lung cancer (NSCLC) patients (Zang et al., 2017). Blockade of VEGF-A by bevacizumab decreases the microvessel structure density in tumors, reduces tumor volumes (Zhao et al., 2012), and reprograms the tumor immune microenvironment (Tamura et al., 2019), thus providing a rationale for combination strategies.

Previous observational cohort studies mainly explored the use of paclitaxel/platinum plus bevacizumab versus paclitaxel/platinum therapy in advanced NSCLC and demonstrated improved response and survival benefits of bevacizumab (Sandler et al., 2006; Zhou et al., 2015). A pointbreak study directly compared pemetrexed or paclitaxel combined with carboplatin and bevacizumab in patients with previously untreated stage IIIB or IV non-squamous NSCLC and reported significantly improved progression-free survival (PFS) but not overall survival (OS) (Patel et al., 2013). However, studies on pemetrexed-based chemotherapy with or without bevacizumab mostly focus on the maintenance therapy (Barlesi et al., 2013; Paz-Ares et al., 2013). The association between clinicopathologic characteristics and treatment outcomes of patients receiving pemetrexed-platinum doublet plus bevacizumab and specific populations which might or might not achieve tumor remission remains unclear.

Therefore, studies on whether pemetrexed-based chemotherapy plus antiangiogenesis agents can indeed prolong patients’ survival with tolerable safety profiles in real-world settings are needed. In this study, we conducted a retrospective real-world study comparing pemetrexed-platinum doublet plus bevacizumab (Bev+PP) with doublet alone (PP) in Chinese chemo-naive advanced lung adenocarcinoma patients.

## Materials and Methods

### Study Design and Patient Enrollment

This retrospective study collected clinical data from patients with advanced lung adenocarcinoma who received at least one cycle of first-line pemetrexed–platinum chemotherapy with or without bevacizumab between April 2014 and June 2020 in our own hospital. The study was approved by the Hangzhou Cancer Hospital Ethics Review Board. This study is observational and presents no more than minimal risk of harm to subjects and involves no procedures for which written consent is normally required outside the research context. The Hangzhou Cancer Hospital Ethics Review Board approved the waiver of informed consents for this study according to section 39 of Measures for the Ethical Review of Biomedical Research Involving Humans published by the National Health Commission of the People’s Republic of China (CLI.4.282697). The inclusion criteria were as follows: 1) pathologic and radiographic confirmation of stage IIIb-IV (AJCC 7th edition) lung adenocarcinoma patients; 2) patients with no history of prior chemotherapy or antiangiogenesis drugs administration; 3) recipients of Bev+PP or PP; 4) recipients of ≥1 cycles of chemotherapy; and 5) complete medical records. Patients were excluded if they 1) were receiving other categories of antitumor therapy during the indicated treatments, 2) were given bevacizumab after the progression of pemetrexed–platinum–based chemotherapy, or 3) were lost to follow-up.

### Data Collection

Patients’ private information remained confidential; however, the characteristics including age, gender, smoking history, baseline Eastern Cooperative Oncology Group Performance Score (ECOG PS), pathological diagnosis and staging, medical history, and imaging data were captured from the electronic health system. The follow-up was conducted using both outpatient and telephone appointments. Treatment responses were assessed as complete response (CR), partial response (PR), stable disease (SD), or progressive disease (PD), by comparing the imaging data before and after treatment using the Response Evaluation Criteria in Solid Tumors (RECIST) version 1.1. The objective response rate (ORR) was calculated as CR + PR/total cases, while the disease control rate (DCR) was calculated as CR + PR + SD/total cases. PFS was defined as the time from treatment initiation to the first confirmation of disease progression or death, while OS was defined as the time from treatment initiation until death. Adverse events were graded according to the National Cancer Institute—Common Toxicity Criteria for Adverse Events version 4.0. Two professional oncologists performed the grading of the treatment responses and adverse events independently, which were later recorded in the database.

### Statistical Analysis

A chi-square test or Fisher’s exact test was used to assess for independence between the categorical variables in the two treatment groups with or without bevacizumab. The Kaplan–Meier method and log-rank test were used to compare the efficacy of combination therapy with chemotherapy alone. Multivariate Cox analyses were employed to estimate the factors affecting the efficacy of treatment (PFS and OS). For matched factors affecting PFS, the patients were divided on that factor and further analyzed whether the strength of one treatment could still be present in the subgroup. All analyses were performed with IBM SPSS Statistics 26.0. Survival curves were drawn using Prism GraphPad 8.0.

## Results

### Clinicopathologic Characteristics

Between April 2014 and June 2020, a total of 100 chemotherapy-naive patients with advanced lung adenocarcinoma at our institute met the inclusion criteria and were enrolled on our retrospective real-world study comparing the efficacy and safety of pemetrexed–platinum with or without bevacizumab (46 patients in the Bev+PP group and 54 patients in the PP group).

The median age was 62 (38–75) years in the Bev+PP group and 63 (18–83) years in the PP group. Bev+PP and PP groups enrolled similar proportions of male (71.7 vs. 59.3%, *p* = 0.2133), elder (60.9 vs. 63.0%, *p* = 0.8298), ever smoking (56.5 vs. 79.6%, *p* = 0.08), ECOG PS = 1 (89.1 vs. 90.7%, *p* = 0.9648), and EAR-negative (56.5 vs. 68.5%, *p* = 0.3418) patients. The occurrence rates of metastasis in organs including the lung, bone, pleural, brain, adrenal glands, and liver, and the EAR gene mutation status were distributed evenly between the two groups (*p* = 0.5052, 0.3418, respectively). However, the Bev+PP group reported more later stage lung adenocarcinoma patients than PP group (IV_b+c_ 67.4 vs. 37.0%, *p* = 0.0066). (Detailed data are shown in Table 1.) The pre- and post-line treatment history information are presented in Supplementary Table S1; in brief, the Bev+PP group enrolled a higher number of patients reporting failed prior TKI treatments than the PP group (26.1 vs. 14.8%, *p* = 0.160).

**TABLE 1:** Clinical characteristics of lung adenocarcinoma patients.

Characteristic	Bev+PP (*n* = 46)		PP (*n* = 54)		*p*-value
	N	%	N	%	
Age (years)	62 [38–75]		63 [18–83]		
≥60	28	60.9	34	63.0	0.8298
<60	18	39.1	20	37.0	
Gender					
Male	33	71.7	32	59.3	0.2133
Female	13	28.3	22	40.7	
Smoking					
Never	20	43.5	11	20.4	0.08
Ever	26	56.5	43	79.6	
ECOG PS					
0	3	6.5	3	5.6	0.9648
1	41	89.1	49	90.7	
2	2	4.3	2	3.7	
Tumor stage					
III_b_	2	4.3	9	16.7	0.0066*
IV_a_	13	28.3	25	46.3	
IV_b+c_	31	67.4	20	37.0	
Site of metastasis					
Lung	17	37.0	20	37.0	0.5052
Bone	20	43.5	21	38.9	
Pleura	11	23.9	15	27.8	
Brain	14	30.4	13	24.1	
Adrenal glands	9	19.6	3	5.6	
Liver	6	13.0	4	7.4	
Driver gene status					
EAR negative	26	56.5	37	68.5	0.3418
EGFR mutation	12	26.1	13	24.1	
ALK or ROS1 fusion	2	4.3	2	3.7	
Unknown	6	13.0	2	3.7	

### Treatment and Efficacy

**FIGURE 1:**
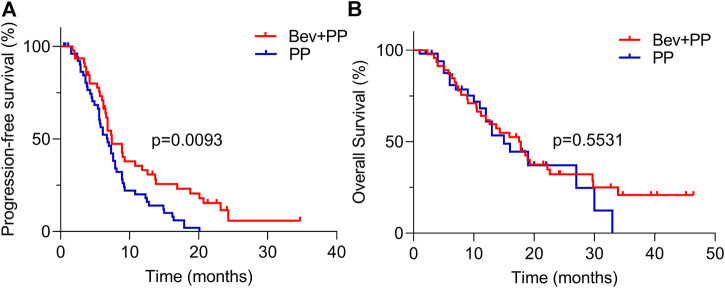
PFS and OS curves of Bev+PP (red) and PP (blue) groups. **(A)** Kaplan–Meier survival curves showing the PFS of patients in the Bev+PP group (*n* = 46) and PP group (*n* = 54) (median PFS, 7.4 vs. 6.8 months; HR [95% CI]: 0.59 [0.39–0.90], *p* = 0.0093); **(B)** Kaplan–Meier survival curves showing the OS of patients in the Bev+PP group (*n* = 46) and PP group (*n* = 54) (median OS, 17.5 vs. 15 months; HR [95% CI]: 0.86 [0.50–1.47], *p* = 0.5531).

In brief, patients were intravenously injected with 500 mg/m2 pemetrexed on d1, 37.5 mg/m2 cisplatin on d1-2, or carboplatin (area under the curve, 4–5) on d1, with or without 7.5 mg/kg bevacizumab on d1 during induction therapy. Pemetrexed plus bevacizumab or pemetrexed monotherapy was administered during maintenance therapy. Patients in the two groups received similar cycles of induction therapy (mean cycle 4.1 [2–7] vs. 4.2 [1–6] for PP and Bev+PP groups, respectively, *p* = 0.9740). However, patients receiving Bev+PP were more likely to receive more cycles of maintenance therapy than patients in the PP group (5.3 [0–22] vs. 2.5 [0–18], *p* = 0.0015). Although none of the patients achieved complete clearance of cancer, 65% of patients in the Bev+PP group had PR, obviously higher than only 30% in the PP group (same with ORR, *p* = 0.0004). And surprisingly, only one patient experienced PD after administration of bevacizumab and pemetrexed–platinum, with the DCR of the Bev+PP group reaching 98% (45/46), compared with 87% in the PP group (*p* = 0.0663) (Table 2). Patients in the Bev+PP group achieved superior PFS than those in the PP group (median PFS, 7.4 vs. 6.8 months; HR [95% CI]: 0.59 [0.39–0.90], *p* = 0.0093) (Figure 1A). However, addition of bevacizumab did not cause any significant improvement in OS (median OS for Bev+PP and PP, 17.5 vs. 15.0 months; HR [95% CI]: 0.86 [0.50–1.47], *p* = 0.5531) (Figure 1B). When we merely looked at EAR-negative patients, bevacizumab addition also tended to improve PFS (median PFS, 8.9 vs. 7.0 months; HR [95% CI]: 0.63 [0.37–1.07], *p* = 0.0836), but not OS (median OS, 17.5 vs. 16.2 months; HR [95% CI]: 0.99 [0.51–1.91], *p* = 0.9638) (Supplementary Figures S1A,B). Multivariate Cox regression analysis showed that treatment with or without bevacizumab (*p* = 0.001), gender (*p* = 0.008), and adrenal metastasis (*p* = 0.001) as well as liver metastasis (*p* = 0.013) were independent risk factors for the PFS in lung adenocarcinoma patients (Table 3), reaffirming that bevacizumab addition improved the PFS of chemo-naive lung adenocarcinoma patients.

**TABLE 2:** Efficacy profile and treatment cycles of the two groups.

	Bev+PP (*n* = 46)	PP (*n* = 54)	*p*-value
	N (%)	N (%)	
Best response			
CR	0 (0)	0 (0)	
PR	30 (65)	16 (30)	
SD	15 (33)	31 (57)	
PD	1 (2)	7 (13)	
ORR	30 (65)	16 (30)	0.0004*
DCR	45 (98)	47 (87)	0.0663
Induction cycle mean (range)	4.1 [2–7]	4.2 [1–6]	0.9740
Maintenance cycle mean (range)	5.3 [0–22]	2.5 [0–18]	0.0015*

**TABLE 3:** Multivariate analysis of PFS in all patients.

Characteristic	*p*-value	HR	95% CI	
			Lower limit	Upper limit
Bev+PP	0.001*	0.42	0.25	0.71
Gender = female	0.008*	0.43	0.23	0.80
Age (years)	0.746	1.00	0.97	1.02
Smoking	0.750	0.89	0.44	1.79
Lung metastasis	0.671	0.89	0.52	1.52
Pleura metastasis	0.488	1.24	0.67	2.30
Adrenal metastasis	0.001*	3.80	1.77	8.18
Bone metastasis	0.981	0.99	0.55	1.81
Brain metastasis	0.188	1.49	0.82	2.71
Liver metastasis	0.013*	2.67	1.23	5.77
Distant metastasis	0.118	0.47	0.18	1.21
ECOG ≥ 2	0.232	2.17	0.61	7.68
EGFR mutation	0.149	1.56	0.85	2.85
ALK/ROS1 fusion	0.998	1.00	0.30	3.36
Unknown status	0.882	0.93	0.38	2.30

The relationship between the baseline clinicopathologic characteristics of enrolled patients and PFS or OS was further analyzed by multivariate Cox analyses to screen out a specific population of patients who could optimally benefit from Bev+PP treatment and risk factors that reduced the efficacy. As listed in Table 4, female patients had superior OS than male patients (Bev+PP, HR [95% CI]: 0.18 [0.04–0.87]; PP, HR [95% CI]: 0.08 [0.01–0.53]). EGFR or ALK/ROS1 status did not affect the efficacy of Bev+PP (*p* = 0.899 and 0.984) on OS. Additionally, adrenal metastasis severely reduce the PFS of patients receiving Bev+PP (HR [95% CI]: 10.17 [2.99–34.62]).

**TABLE 4:** Multivariate Cox regression analysis of OS and PFS in the Bev+PP group.

Category	OS		PFS	
	HR	*p*-value	HR	*p*-value
Gender = female	0.18 [0.04–0.87]	0.032*	0.25 [0.07–0.90]	0.034*
Age (years)	1.00 [0.95–1.06]	0.899	0.99 [0.95–1.04]	0.796
Smoking	0.81 [0.23–2.77]	0.731	0.85 [0.28–2.64]	0.784
Distant metastasis	0.95 [0.09–9.83]	0.65	0.82 [0.08–8.33]	0.65
Lung metastasis	2.23 [0.79–6.27]	0.130	1.57 [0.65–3.82]	0.318
Pleura metastasis	0.51 [0.17–1.58]	0.245	0.58 [0.22–1.54]	0.273
Adrenal metastasis	2.18 [0.70–6.82]	0.179	10.17 [2.99–34.62]	0.000*
Bone metastasis	0.90 [0.36–2.25]	0.813	0.77 [0.33–1.81]	0.551
Brain metastasis	0.62 [0.24–1.62]	0.329	1.50 [0.59–3.85]	0.395
Liver metastasis	4.34 [0.99–18.91]	0.051	3.04 [0.88–10.58]	0.080
ECOG ≥ 2	1.10 [0.16–7.49]	0.919	2.27 [0.33–15.39]	0.402
EGFR mutation	1.08 [0.33–3.56]	0.899	1.28 [0.43–3.78]	0.660
ALK/ROS1 fusion	0.00 [0.00-NA]	0.984	0.69 [0.09–5.38]	0.720
Unknown status	0.28 [0.06–1.25]	0.095	0.34 [0.09–1.32]	0.118

### Subgroup Analysis

**FIGURE 2:**
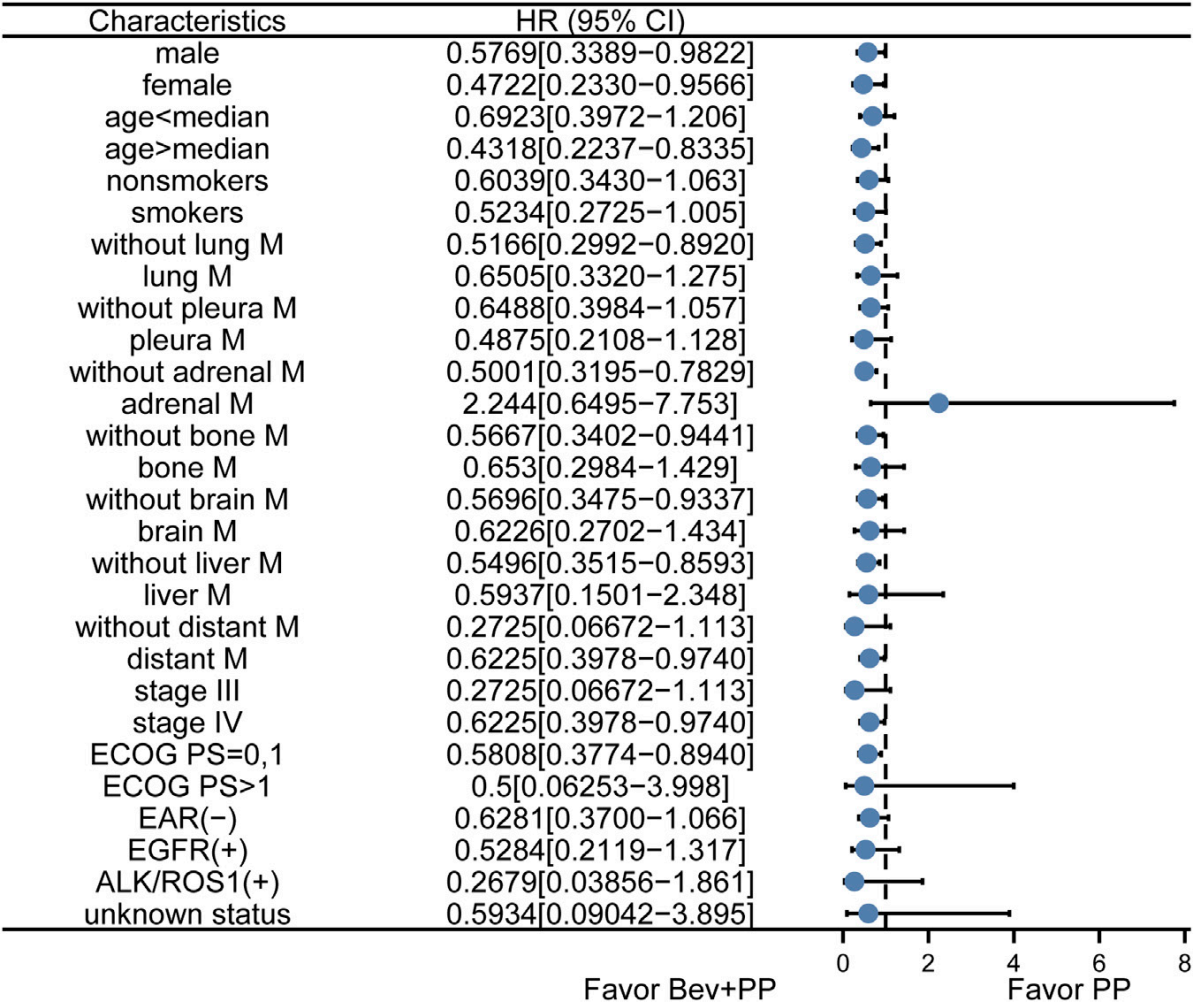
Subgroup analysis of PFS in enrolled patients. Dots and horizontal lines represent the mean value and confidence intervals, respectively. Bev+PP denotes bevacizumab plus pemetrexed–platinum, and PP denotes pemetrexed–platinum.

To further confirm whether adrenal metastasis or other characteristics may favor Bev+PP or PP on PFS, we conducted a subgroup analysis of hazard ratios for progression of enrolled patients after Bev+PP or PP administration using the Kaplan–Meier method. As shown in Figure 2, the effect of bevacizumab on the pemetrexed-platinum chemotherapy was generally consistent in subgroups including gender, age, smoking, stage, distant metastasis, and EAR status. However, adrenal metastasis tended to be relatively in favor of pemetrexed–platinum doublet alone (HR [95% CI]: 2.244 [0.6495–7.753]).

### Adverse Events

The prevalence of all adverse events (AEs), including grade ≥3 adverse events in the two groups was similar (all AEs, *p* = 0.465; all grade ≥3 AEs, *p* = 0.652). The top three frequent AEs of patients in Bev+PP and PP groups were leukopenia (63.0%, 59.3%), fatigue (45.7%, 38.9%), and nausea (37.0%, 33.3%) (Table 5). Grade 3–4 leukopenia was the most common grade ≥3 AE occurring in 19.6 and 18.5% of patients in Bev+PP and PP groups, respectively, while other grade ≥3 AEs were rare in the two groups. The safety profiles in both groups were acceptable, and no new unexpected AEs were observed.

**TABLE 5:** Safety profile of the two groups.

	All AEs			Grade ≥3 AEs		
	Bev+PP (*n* = 46)	PP (*n* = 54)	*p*-value	Bev+PP (*n* = 46)	PP (*n* = 54)	*p*-value
	N (%)	N (%)		N (%)	N (%)	
Leukopenia	29 (63.0)	32 (59.3)	0.837	9 (19.6)	10 (18.5)	0.999
Abnormal liver function	12 (26.1)	9 (16.7)	0.326	1 (2.2)	2 (3.7)	0.999
Fatigue	21 (45.7)	21 (38.9)	0.546	1 (2.2)	2 (3.7)	0.999
Nausea	17 (37.0)	18 (33.3)	0.834	0 (0.0)	1 (1.9)	0.999
Anemia	12 (26.1)	14 (25.9)	0.999	1 (2.2)	3 (5.6)	0.622
Thrombocytopenia	10 (21.7)	8 (14.8)	0.438	1 (2.2)	0 (0.0)	0.460
Thrombosis and hemorrhage	2 (4.3)	1 (1.9)	0.593	1 (2.2)	0 (0.0)	0.460
Hypertension	2 (4.3)	0 (0.0)	0.209	0 (0.0)	0 (0.0)	NA
Anorexia	2 (4.3)	5 (9.3)	0.447	0 (0.0)	0 (0.0)	NA
Headache	1 (2.2)	0 (0.0)	0.460	0 (0.0)	0 (0.0)	NA
Rash	1 (2.2)	1 (1.9)	0.999	0 (0.0)	0 (0.0)	NA
Increased creatinine	2 (4.3)	0 (0.0)	0.209	0 (0.0)	0 (0.0)	NA
Total	41 (89.1)	51 (94.4)	0.465	11 (23.9)	16 (29.6)	0.652

## Discussion

In the current real-world study, the addition of bevacizumab in first-line pemetrexed–platinum (either cisplatin or carboplatin) doublet significantly improved PFS compared to doublet alone, with similar and tolerable adverse events, although no differences in OS was observed between the two groups. Multivariate Cox regression analyses confirmed that pemetrexed–platinum doublet with bevacizumab was an independent favorable factor of PFS. This study confirmed that pemetrexed–platinum doublet combined with bevacizumab was safe and effective for Chinese chemo-naive lung adenocarcinoma patients.

Bevacizumab plus pemetrexed–platinum doublet chemotherapy has become a standard first-line treatment for advanced lung adenocarcinoma patients with negative results of detecting PD-L1 expression, EGFR mutations, and ALK/ROS1 gene fusions, or after the failure of targeted therapies for patients with EGFR mutations or ALK/ROS1 fusions, as recommended in NCCN NSCLC guidelines (Ettinger et al., 2019) and pan-Asian adapted ESMO NSCLC guideline (Wu et al., 2019), based on several large phase III clinical trials, including SAiL and ARIES (Crinò et al., 2010; Lynch Jr et al., 2014) and several other clinical trials comparing paclitaxel-based chemotherapy with or without bevacizumab. Consistent with the ORR and PFS benefits of bevacizumab addition found in these clinical trials, our study also demonstrated similar PFS and ORR improvements.

AVAPERL (Barlesi et al., 2013) and COMPASS (Seto et al., 2020) studies compared pemetrexed plus bevacizumab with single-agent bevacizumab in the maintenance and whole phases, respectively, which showed elevated PFS but not OS with bevacizumab addition. A Japanese clinical trial published in 2016 reported a nonsignificant beneficial PFS in the pemetrexed–platinum plus bevacizumab group compared with pemetrexed–platinum alone (mPFS 11.5 vs. 7.3 months, *p* = 0.198) (Karayama et al., 2016). The 2016 Japanese study and another Japanese study SAKK19_09 (Gautschi et al., 2015) all reported no significant improvement in OS when comparing Bev+Pem–platinum with chemo-monotherapy (24.4 vs. 21.3 months, *p* = 0.63; 14.7 vs. 14.6 months, *p* = 0.890; respectively). However, Stephen J et al. conducted a large sample retrospective cohort study including 4,724 patients with 58% receiving carboplatin–pemetrexed and 42% receiving carboplatin–pemetrexed–bevacizumab. The study demonstrated an OS benefit from 8.6 to 12.1 months (*p* < 0.001) and also showed that female patients had longer OS than male patients after the triple-reagent administration (Bagley et al., 2019). In our study, more patients received longer maintenance cycles of pemetrexed plus bevacizumab treatment, which might have contributed to the longer PFS and also indicated that the safety profile of combination therapy was tolerable in the real-world setting. Although the addition of bevacizumab or not was a constant factor in both induction and maintenance therapies, the final OS was not significantly altered in our study, which was consistent with findings from the abovementioned clinical studies but not the large-sample retrospective cohort study. We also found that female patients treated with pemetrexed–platinum with bevacizumab responded better than male patients. The limited PFS and insignificant OS improvement in our study might be due to a higher percent of patients with stage IV b+c lung adenocarcinoma. Besides, the limited population size may also cause the restricted application of our findings, and uncontrolled post-line treatments in the real-world may have an impact on the OS.

Several other studies have also reported the use of combination therapies in real-world settings. For instance, Katherine B. Winfree retrospectively compared maintenance therapies for non-squamous lung cancer patients in the United States and found that pemetrexed–bevacizumab treatment could improve patients’ PFS compared to pemetrexed treatment alone (Winfree et al., 2019). However, the PFS was calculated from the first day of induction therapy, while the induction therapies included many different regimens such as platinum + bevacizumab, and platinum + other chemo-reagents. Thus, the improvement may be all attributed to the specific maintenance regimen. In our study, we specifically focused on pemetrexed–platinum doublet with or without bevacizumab, and the comparison was intended to investigate whether the addition of bevacizumab could benefit Chinese chemo-naive lung adenocarcinoma patients during the entire treatment, and we did observe an improvement in PFS but not in OS. Fei Qi et al. (Qi et al., 2019) and Xiaoyou Li et al. (Li et al., 2019) performed similar comparisons with ours and also found that the median PFS was significantly prolonged in the bevacizumab addition group compared with the chemo-only group (9.8 vs. 7.8 months, *p* = 0.006; 10.97 vs. 6.67 months, *p* = 0.0002), as well as ORR, which were consistent with our findings. More than 4 months of PFS improvement may not be all attributed to bevacizumab addition since the combination group enrolled significantly fewer patients with brain or pleural metastasis than the chemo-group, whereas in our study, a higher percent of patients enrolled in the pemetrexed–platinum treatment group were absent of distant metastasis, than the combination group.

The MAP study showed that when receiving platinum/pemetrexed/bevacizumab, patients with EGFR mutation had higher OS than patients with EGFR-negative status (NR vs. 20.7 months, *p* = 0.004). However, only 8 out of 23 patients had prior TKIs (Tsutani et al., 2018). The 2016 Japanese study also enrolled patients with failure from EGFR or ALK/ROS1 tyrosine kinase inhibitors; however, the authors failed to compare the survival difference between negative and positive patients (Karayama et al., 2016). EGFR-negative patients in the COMPASS study obtained a better OS with bevacizumab addition (HR [95% CI], 0.82 [0.68–0.99]) (Seto et al., 2020). In this study, we assessed the treatment efficacy in subgroups using outcome PFS, but not OS because the OS of the total samples did not show any significant tendency to either treatment. Bevacizumab addition tended to be beneficial to both EGFR-negative and -positive patients.

Our study also compared combination therapy with pemetrexed–platinum doublet in subgroup analysis, and surprisingly, the findings demonstrated that it tended to be favoring chemotherapy alone for patients with adrenal metastasis. However, to the best of our knowledge, no clinical trials or other retrospective studies analyzing such a specific population have been reported. Only the ECOG4599 study comparing paclitaxel–carboplatin doublet plus bevacizumab with chemotherapy alone in NSCLC reported the hazard ratio for OS (0.97 [0.65–1.46]) of patients with adrenal metastasis, with the largest value in analyses of all sites of metastasis, indicating the highest risk not benefiting from the addition of bevacizumab (Sandler et al., 2006). We have to admit that limited patients with adrenal metastasis were enrolled in our study, which might cause false discovery. However, to avoid the economic burden of bevacizumab addition, we cautiously suggest that clinical trials or retrospective studies with a large sample size be conducted to further investigate the effect of bevacizumab addition in patients with adrenal metastasis.

The combination treatment was well-tolerated, and no unexpected findings were reported in our real-world study. The two groups shared similar adverse effects, including severe adverse effects. Leukopenia (also categorized as neutropenia in clinical trials), fatigue, and nausea were found to be the most frequent AEs in the two groups, which are reported as common adverse effects of chemotherapy treatment (Zinner and Herbst, 2004), and bevacizumab addition in this study did not significantly augment those side effects. The ECOG-ACRIN 5508 study investigating the strategies for maintenance therapies revealed that the incidence of grade ≥3 AEs was obviously elevated in the combination regimen, from 37 to 51% (Ramalingam et al., 2019). However, such an increase in the incidence of grade ≥3 AEs was not demonstrated in the abovementioned clinical trials and retrospective studies and in the current study. Besides, patients in the ECOG-ACRIN 5508 study were administered with paclitaxel–carboplatin plus bevacizumab as induction therapy before randomization into pemetrexed or pemetrexed plus bevacizumab groups, which might cause late impact on grade ≥3 AEs during maintenance therapy.

In conclusion, although the survival benefit associated with bevacizumab addition is reported limited in our study, the overall response is increased and the safety profile is acceptable. The addition of bevacizumab is currently recommended and has been demonstrated to be a better treatment option in chemo-naive lung adenocarcinoma patients with PD-L1–negative scores in many clinical trials and real-world studies including this study. However, we carefully suggest that medical oncologists should be cautious with bevacizumab addition in patients with adrenal metastasis, unless large clinical studies focusing on this specific population are performed and show clear evidence.

## Data Availability

The raw data supporting the conclusions of this article will be made available by the authors, without undue reservation.

## Supplementary Material

Supplementary Figure S1 | PFS and OS curves of EAR negative patients in Bev+PP (red) and PP 418 (blue) groups. (A) Kaplan-Meier survival curves showed the PFS of EAR negative patients in Bev+PP 419 group (n = 26) and PP group (n = 37) (median PFS, 8.9 vs. 7.0 months; HR[95%CI]: 0.63[0.37–1.07], 420 p = 0.0836); (B) Kaplan-Meier survival curves showed the OS of EAR negative patients in Bev+PP 421 group (n = 26) and PP group (n = 37) (median OS, 17.5 vs. 16.2 months; HR[95%CI]: 0.99[0.51–1.91], 422 p = 0.9638).
