# Supplementary material for: Pemetrexed-Platinum With or Without Bevacizumab for Chinese Chemo-Naive Advanced Lung Adenocarcinoma Patients: A Real-World Study
Source: Front Pharmacol. 2021 May 7;12:649222. doi: 10.3389/fphar.2021.649222 (PMC8138310; doi:10.3389/fphar.2021.649222)
Supplement: Supplementary file 1 [file Table1.DOCX]

| Treatment history | Bev+PP(46) | | PP(54) | | | p-value  (chi-square test) |
| --- | --- | --- | --- | --- | --- | --- |
|  | N % | | N % | | |  |
| Without Prior TKIs | 34 | 73.9% | | 46 | 85.2% | 0.160 |
| With Prior TKIs | 12 | 26.1% | | 8 | 14.8% |  |
| 1G TKIs | 9 | 19.6% | | 8 | 14.8% |  |
| 1G+3G TKIs | 3 | 6.5% | | 0 | 0.0% |  |
| With Post-line TKIs | 10 | 21.7% | | 8 | 14.8% | 0.369 |
| Without Post-line TKIs | 36 | 78.3% | | 46 | 85.2% |  |
| Post-line Chemo <2 lines | 14 | 30.4% | | 18 | 33.3% |  |
| Post-line Chemo ≥2 lines | 3 | 6.5% | | 4 | 7.4% |  |
| Post-line Immunotherapy | 3 | 6.5% | | 4 | 7.4% |  |

**Supplementary table 1.** The prior or post line treatment history of the two groups
