# Supplementary figures and images for: Pemetrexed-Platinum With or Without Bevacizumab for Chinese Chemo-Naive Advanced Lung Adenocarcinoma Patients: A Real-World Study
Source: Front Pharmacol. 2021 May 7;12:649222. doi: 10.3389/fphar.2021.649222 (PMC8138310; doi:10.3389/fphar.2021.649222)

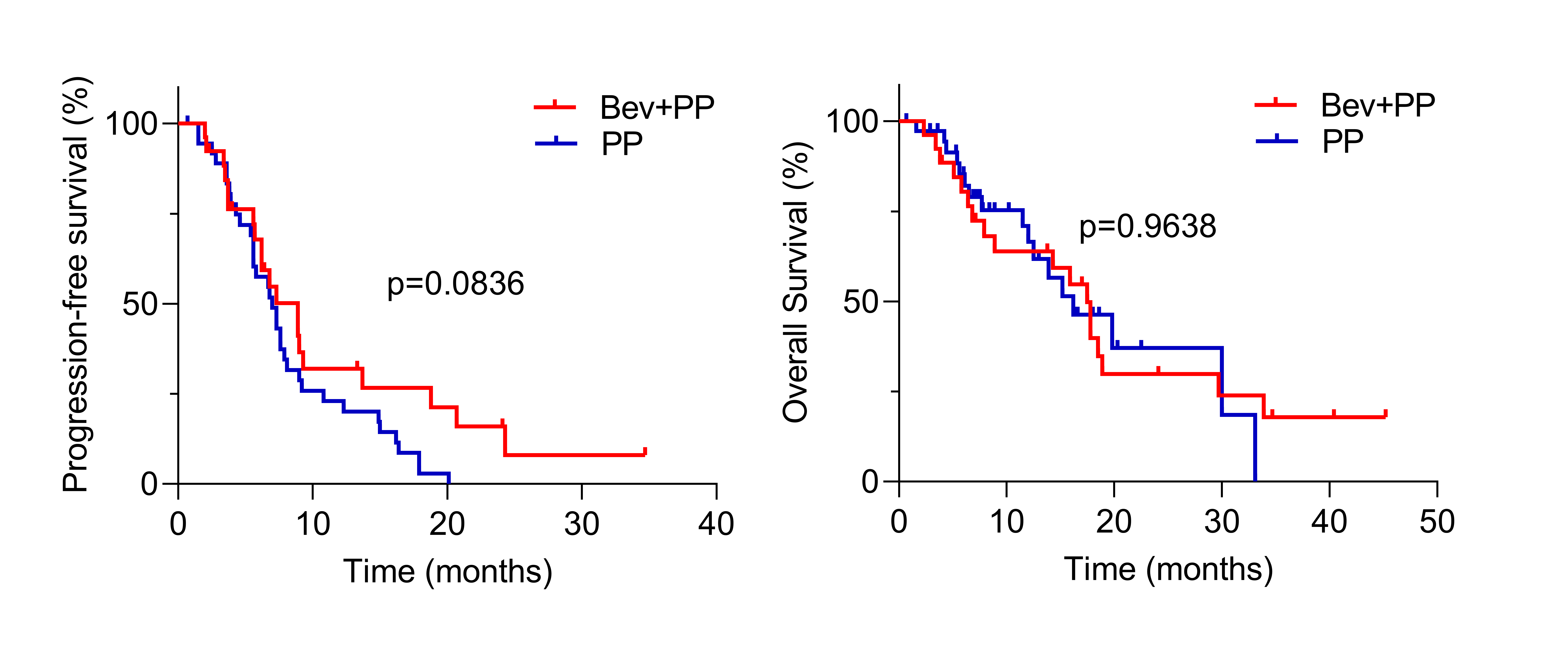

Supplement: Supplementary file 2 [file Image1.TIF]
